# A rare case of progressive left parotid sialolipoma with parapharyngeal extension: case report

**DOI:** 10.1093/jscr/rjaf1077

**Published:** 2026-01-18

**Authors:** Moaaz Amir, Salwa Sheikh

**Affiliations:** Imam Abdulrahman Bin Faisal University, College of Medicine, Eastern Province, Dammam 31441, Saudi Arabia; Johns Hopkins Aramco Healthcare, Department of Pathology, Eastern Province, Dhahran 34465, Saudi Arabia

**Keywords:** Sialolipoma, salivary, parotid, tumor

## Abstract

Sialolipomas are uncommon benign lipomatous neoplasm of the salivary glands, representing under 1% of parotid tumors. They are typically indolent and asymptomatic, often resembling simple lipomas on imaging. We report the case of a 54-year-old male presented with an asymptomatic enlargement in the left parotid region incidentally discovered. Magnetic resonance imaging displayed a well-circumscribed lipomatous growth sized at approximately 4.6 cm, with incremental growth to 6 cm over three years and involvement of the parapharyngeal space. Due to continued growth and cosmetic concerns, a complete left parotidectomy was carried out with preservation of the facial nerve. Histopathology affirmed sialolipoma. Recognition of this rare entity and its distinction from other similar lipomatous masses, including lipoadenoma, is vital for accurate diagnosis. Full resection yields an excellent prognosis, with recurrence being exceedingly rare.

## Introduction

Lipomas constitute a large portion of soft tissue tumors; however, lipomatous tumors emerging from the salivary glands are rare, representing fewer than 1% of all parotid gland tumors [1]. Sialolipoma is an uncommon histological variant of lipoma defined by the presence of both fully mature adipose tissue and typical salivary gland elements [1, 2]. In practice, sialolipomas are often present as asymptomatic, slow-growing masses and are commonly mistaken for classic lipomas or pleomorphic adenomas in radiologic studies [2, 3]. They may arise in both major and minor salivary glands, most often affecting the parotid gland [1, 3]. Due to the unusual nature of this lesion, the majority of cases are diagnosed postoperatively via pathological analysis [2, 4]. In this report, we describe a case of a left parotid sialolipoma observed radiologically for three years before operative intervention. This case emphasizes the significance of serial imaging, the impact of surgical decision-making in benign yet growing lesions, and the pathological features establishing the diagnosis.

## Case presentation

**Figure 1:**
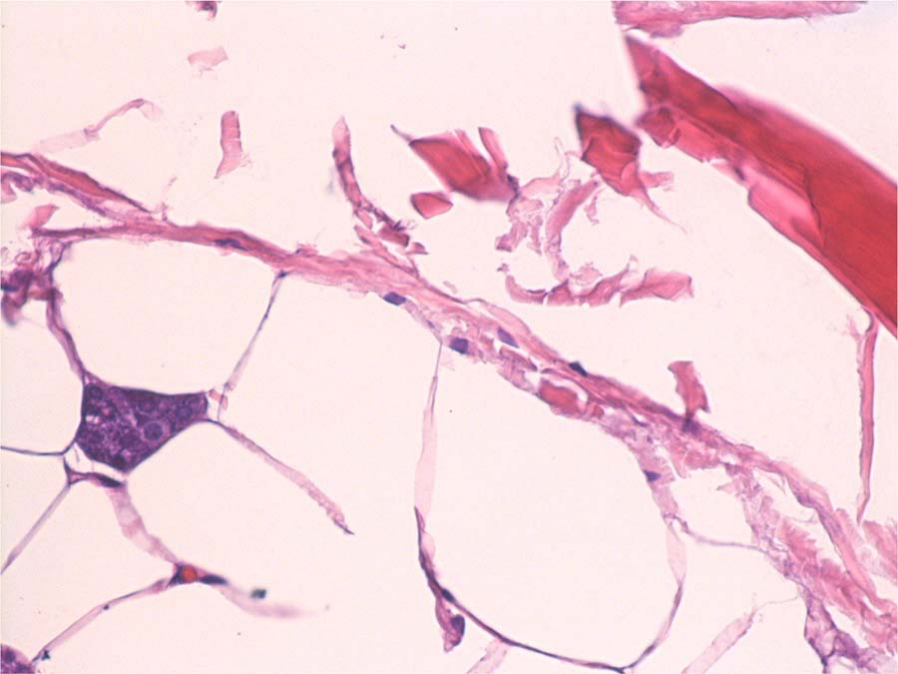
Small groups of acini, randomly distributed in lipomatous tumor.

**Figure 2:**
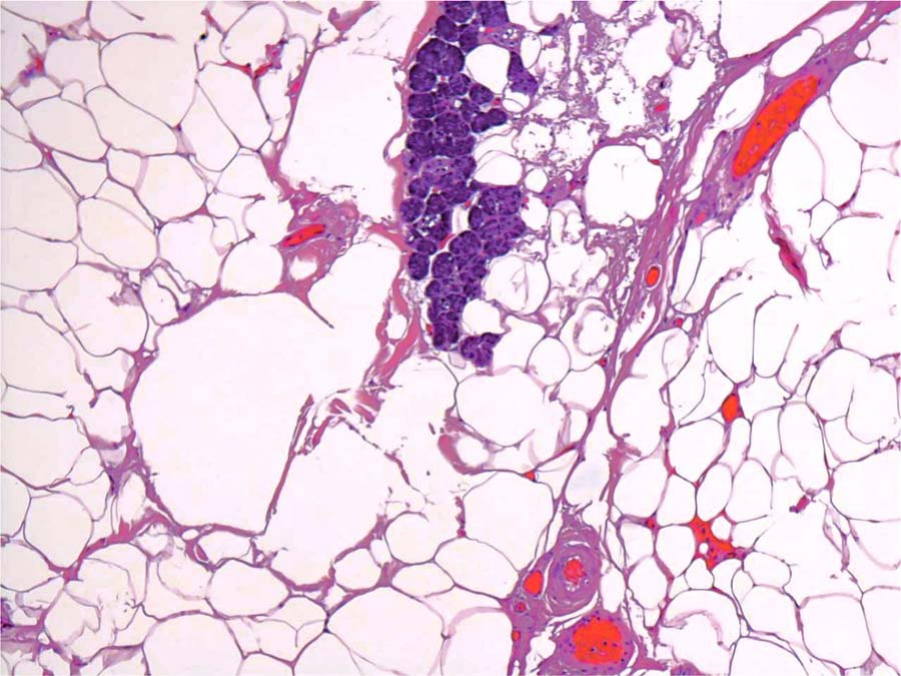
Groups of acini and occasional ducts present within the lipomatous tumor.

A 54-year-old male presented for assessment of a non-tender swelling in the left parotid region. His past medical history was unremarkable. Neck magnetic resonance imaging (MRI) revealed a well-defined adipose lesion in the left parotid gland measuring nearly 4.6 cm in its largest dimension. Neck ultrasound (US) additionally revealed bilateral thyroid nodules; Fine-needle aspiration of the right thyroid nodule confirmed benign follicular changes (Bethesda class II). Follow-up imaging (MRI and US) 1 year later showed a stable parotid lesion measuring 5.3 × 3.2 × 2.8 cm without contrast uptake. Bilateral thyroid nodules were unchanged. The patient was asymptomatic but reported cosmetic worries. Repeat imaging MRI demonstrated mild progression to 4.8 cm, expanding toward the parapharyngeal space. The lesion displayed uniform fat signal with no malignant enhancement. In view of gradual enlargement and patient cosmetic concern, surgical excision was recommended. A complete left parotidectomy with full general anesthesia was performed. During surgery, a lobulated, soft tumor measuring 6 × 4.5 × 2 cm was excised, while sparing all facial nerve branches. No complications were noted. Microscopic examination revealed a lobulated fibro-fatty tumor consisting of well-differentiated adipose tissue with intermixed salivary gland acini and ducts (Figs 1 and 2), establishing the diagnosis of sialolipoma. Postoperative recovery occurred without complications, and the patient showed normal facial nerve function at follow-up.

## Discussion

Sialolipomas are exceedingly uncommon, with under 50 well-documented cases described in the literature [1–3, 7, 8]. Their pathogenesis is not well understood; some researchers suggest they result from adipose infiltration of salivary tissue following chronic inflammation or age-related degeneration, whereas others classify them as a true neoplastic process [2, 4]. Radiologically, sialolipomas resemble simple lipomas owing to their uniform adipose content, but subtle distinctions may exist [3]. MRI usually demonstrates a well-circumscribed lesion exhibiting fat-equivalent intensity on every sequence [3, 5]. The absence of internal contrast and the presence of a thin delicate capsule support a benign etiology [5]. Nevertheless, imaging alone cannot definitively differentiate sialolipomas from other lipomatous tumors such as liposarcoma or fibrolipoma, rendering pathological verification essential [4, 6]. During histopathological evalution of lipomatous salivary gland tumors, distinguishing sialolipoma from lipoadenoma is important to ensure proper classification [4]. Based on the World Health Organization classification of head and neck tumors, lipoadenoma is placed under salivary gland adenomas because of its well-structured epithelial and ductal growth within the adipose tissue [4]. Sialolipoma, on the other hand, is categorized under lipomatous salivary gland lesions, defined by well-differentiated adipose tissue encasing typical salivary acini and ducts with a lobular architecture [1, 2, 4]. While both entities exhibit overlapping characteristics on imaging and gross appearance, lipoadenomas display a more structured adenomatous framework, whereas sialolipomas are mainly fat-based expansions with secondary glandular inclusion [1, 4]. This difference is mainly classificatory, since both lesions are benign and have favorable prognosis following complete resection [4–7]. In this case, the mass underwent surveillance for over three years, displaying gradual but modest growth from 4.6 cm to 6 cm and reaching to the parapharyngeal space without malignant features. The patient’s primary concern was cosmetics, although continued enlargement also played a role in advising operative intervention. Surgical management is dictated by the tumor’s location and involvement of adjacent structures. For parotid lesions, either superficial or total parotidectomy with sparing of the facial nerve is the standard approach [5–7]. In this case, a total parotidectomy was performed due to the tumor’s dimensions and deep lobe extension. Postoperative recovery was smooth, and histology verified the diagnosis of sialolipoma. The prognosis of sialolipoma is very good, with total resection being curative [6–8]. Recurrence is extremely infrequent and typically tied with incomplete resection [5–7]. There are no documented instances of malignant transformation [7, 8].

## Conclusion

This case illustrates the benign nature of parotid sialolipomas and emphasizes the importance of extended radiologic surveillance in lipomatous salivary gland lesions. While many remain unchanged and asymptomatic, progressive enlargement or cosmetic-related worries warrant surgical removal. Histopathological examination continues to be the gold standard for diagnosis, and parotidectomy with facial nerve preservation yields outstanding outcomes [5–8]. Reporting such findings expands the scarce data and assists clinicians in distinguishing sialolipomas from other parotid masses, ensuring proper treatment decisions.

## Data Availability

The data used to support the findings of this study are included within the article.
